# Peak Weight and Height Velocity to Age 36 Months and Asthma Development: The Norwegian Mother and Child Cohort Study

**DOI:** 10.1371/journal.pone.0116362

**Published:** 2015-01-30

**Authors:** Maria C. Magnus, Hein Stigum, Siri E. Håberg, Per Nafstad, Stephanie J. London, Wenche Nystad

**Affiliations:** 1 Department of Chronic Diseases, Division of Epidemiology, Norwegian Institute of Public Health, Oslo, Norway; 2 Department of Community Medicine, Medical Faculty, University of Oslo, Oslo, Norway; 3 Institute Management and Staff, Norwegian Institute of Public Health, Oslo, Norway; 4 Epidemiology Branch, Division of Intramural Research, National Institute of Environmental Health Sciences, National Institutes of Health, Department of Health and Human Services, Research Triangle Park, North Carolina, United States of America; Université Paris Descartes; AP-HP, Groupe Hospitalier Cochin-Saint-Vincent-de-Paul, FRANCE

## Abstract

**Background:**

The immediate postnatal period is the period of the fastest growth in the entire life span and a critical period for lung development. Therefore, it is interesting to examine the association between growth during this period and childhood respiratory disorders.

**Methods:**

We examined the association of peak weight and height velocity to age 36 months with maternal report of current asthma at 36 months (n = 50,311), recurrent lower respiratory tract infections (LRTIs) by 36 months (n = 47,905) and current asthma at 7 years (n = 24,827) in the Norwegian Mother and Child Cohort Study. Peak weight and height velocity was calculated using the Reed1 model through multilevel mixed-effects linear regression. Multivariable log-binomial regression was used to calculate adjusted relative risks (adj.RR) and 95% confidence intervals (CI). We also conducted a sibling pair analysis using conditional logistic regression.

**Results:**

Peak weight velocity was positively associated with current asthma at 36 months [adj.RR 1.22 (95%CI: 1.18, 1.26) per standard deviation (SD) increase], recurrent LRTIs by 36 months [adj.RR 1.14 (1.10, 1.19) per SD increase] and current asthma at 7 years [adj.RR 1.13 (95%CI: 1.07, 1.19) per SD increase]. Peak height velocity was not associated with any of the respiratory disorders. The positive association of peak weight velocity and asthma at 36 months remained in the sibling pair analysis.

**Conclusions:**

Higher peak weight velocity, achieved during the immediate postnatal period, increased the risk of respiratory disorders. This might be explained by an influence on neonatal lung development, shared genetic/epigenetic mechanisms and/or environmental factors.

## INTRODUCTION

Asthma is the most common chronic disease during childhood [1]. Early childhood wheezing symptoms are often linked to lower respiratory tract infections (LRTIs) [2]. Restricted intrauterine growth [3,4], small size at birth [5,6] and higher adiposity during childhood are positively associated with asthma [7–10]. Previous studies with anthropometric measurements throughout childhood report positive [11–16], inverse [17] and null associations [18] between weight increase and asthma. A recent meta-analysis further report a positive association of absolute weight gain between birth and 12 months with asthma [19]. Most previous studies report no association between length increase and asthma [11–14]. No previous study of postnatal growth and asthma has included an evaluation of LRTIs.

Neonatal lung function increases with birth weight and birth length [20]. Neonatal lung function is also inversely associated with asthma development [21]. Plausible mechanisms underlying an association between early childhood growth and respiratory disorders include growth factors influencing lung development [22,23], shared genetic and epigenetic mechanisms [24,25], in addition to common environmental influences such as for example infant feeding [26,27].

In contrast to birth weight and childhood growth trajectories, less research exists on growth during the immediate postnatal period and respiratory disorders. This immediate postnatal period is the period of the fastest growth in the entire life span and a critical period for lung development [22]. Only one study has previously evaluated peak height and weight velocity, usually reached within the first month of life, in relation to asthma [13]. Peak height and weight velocity has been associated with fetal growth in a study from the Netherlands [28]. In the same study, peak weight velocity was also positively associated with childhood obesity [28]. As both fetal growth and childhood obesity is associated with asthma, further information regarding the associations of peak weight and height velocity with respiratory disorders seems valuable.

The objective of the current study was to examine the associations of peak weight and height velocity to age 36 months with development of recurrent LRTIs and asthma. A comparison between discordant sibling pairs adjusts for confounding by shared genetic and environmental factors. Differences between a standard and a sibling pair analysis may therefore indicate confounding by factors that are shared by siblings [29]. The current study therefore also incorporated a sibling pair comparison when examining the association of early childhood growth with respiratory disorders.

## MATERIALS AND METHODS

### Study subjects

The study population was the Norwegian Mother and Child Cohort Study (MoBa), which is a population-based pregnancy cohort in Norway [30,31]. MoBa recruited pregnant women at 18 gestational weeks across Norway between 1999 and 2008. The participation rate was 40.6%. Mothers could participate with multiple pregnancies, resulting in approximately 95,200 mothers and 114,500 children. We used data available around March 2014, comprising 114,761 children. MoBa was linked to the Medical Birth Registry of Norway (MBRN). Children not linked to the MBRN (n = 499) and multiple births (n = 3,971) were not eligible for the current study. We used information from questionnaires at 18 and 30 gestational weeks, and when the child was 6-, 18-, 36- months and 7 years. The study is reported according to the Strengthening the Reporting of Observational Studies in Epidemiology (STROBE) recommendations.

### Ethics statement

The Norwegian Data Inspectorate and the Regional Ethics Committee for Medical Research for South/East Norway approved this study. All participants in MoBa gave written informed consent.

### Exposure

Birth weight and height was gathered from the MBRN. Mothers reported anthropometric measurements on their child’s health report cards as recorded by public health nurses according to guidelines from the Norwegian Directorate of Health at 6 weeks and 3, 6, 8, 12, 15–18, 24 and 36 months [32]. The mother also reported the child’s weight and height at 7 years. Growth curves for the child’s growth the first 36 months of life were fitted using the Reed1 model for each gender separately: Y = A + Bt + Cln(t+30) + D/(t+30) [33–35]. The Reed1 model is described in more detail in (S1 Text). The Reed1 model fits growth the first 36 months among MoBa children best compared to other well-known growth models [36]. We decided a priori that the exposures of interest were the peak weight and height velocity as calculated using the first derivative of the Reed1 model. We used multilevel mixed-effects linear regression to implement the Reed1 model among children with a minimum of three postnatal anthropometric measurements.

### Outcome

We defined current asthma at 36 months by maternal report of current asthma in combination with having used an inhaled asthma medication in the past year. Inhaled asthma medications were extracted from an open ended question which asked the mother to list the names of all medications the child had taken and included glucocorticoids and/or beta-2 agonists. LRTIs included maternal report of pneumonia, bronchitis, and/or respiratory syncytial virus when the child was 6 and 18 months and maternal report of pneumonia and/or bronchitis at 36 months. Mothers reported frequency of LRTIs by 6 months, between 6 and 18 months, and between 18 and 36 months. We classified recurrent LRTIs by 36 months as three or more LRTIs. Current asthma at 7 years was defined by maternal report of asthma symptoms the past year in combination with having used an asthma medication the past year. The mother responded either yes or no to a closed ended question regarding whether the child had used any medications for asthma the past year when the child was 7 years.

### Covariates

Potential confounders included characteristics that might influence the child’s growth and development of respiratory disorders. We illustrated this through a directed acyclic graph (DAG; S1 Fig.). Maternal characteristics included age, parity, education, salary, smoking during pregnancy and folate intake during pregnancy [37–39]. Characteristics indicating genetic predisposition included maternal and paternal length, body mass index (BMI) and asthma. Child characteristics included gender, gestational age and whether the child was given breast milk as the only source of milk the first 6 months. Covariates were categorized as indicated in Table 1.

**Table 1:** Distribution of characteristics among individuals with follow-up information available for the analysis of asthma at 36 months and current asthma at 7 years.

**Characteristic**	**Category**	**Sample current asthma at 36 months (N = 50,311)**	**Sample current asthma at 7 years (N = 24,827)**
**Maternal age at delivery** (Mean (SD))	NA	30.4 (4.4)	30.5 (4.3)
**Maternal parity** (%)	Primiparous	48.2	45.8
	1	33.9	34.9
	2	14.1	15.1
	3 or more	3.8	4.2
**Maternal education** (%)	Less than high school	5.3	5.4
	High school	26.6	27.7
	Up to 4 years of college	43.3	44.9
	More than 4 years of college	24.8	22.0
**Maternal yearly salary**(%)	<200,000 NOK	25.8	27.9
	200–400,000 NOK	61.8	62.6
	>400,000 NOK	12.5	9.5
**Maternal height in cm** (Mean (SD))	NA	168.3 (5.9)	168.3 (5.9)
**Maternal pre-pregnancy BMI** (%)	Underweight (<18.5)	2.9	2.7
	Normal weight (18.5–24.9)	66.9	66.7
	Overweight (25–29.9)	21.6	22.1
	Obese (> = 30)	8.6	8.5
**Paternal height in cm** (Mean (SD))	NA	181.6 (6.4)	181.6 (6.4)
**Paternal BMI** (%)	Underweight (<18.5)	0.2	0.2
	Normal weight (18.5–24.9)	46.1	46.2
	Overweight (25–29.9)	45.5	45.6
	Obese (> = 30)	8.3	8.0
**Maternal smoking during pregnancy** (%)	No	92.7	92.5
	Yes	7.3	7.5
**Maternal folate intake during pregnancy** (%)	None	12.7	14.2
	Only first trimester	24.1	21.8
	Only after first trimester	8.9	10.6
	Both first trimester and after	54.3	53.3
**Maternal asthma** (%)	No	92.9	93.0
	Yes	7.1	7.0
**Paternal asthma**(%)	No	91.2	91.5
	Yes	8.9	8.5
**Child gender**(%)	Male	51.1	51.2
	Female	48.9	48.8
**Child gestational age in weeks** (Mean (SD))	NA	39.5 (1.7)	39.5 (1.7)
**Breastfeeding 0–6 months**(%)^a^	None	2.5	2.5
	Partial	52.7	51.7
	Exclusive	44.8	45.8

SD: standard deviation, BMI: body mass index

The amount of missing information on individual covariates was generally low (<2%).

^a^ Breastfeeding the first 6 months characterized as partial if the mother reported giving the child breast milk and another source of milk (i.e. formula). Breastfeeding characterized as exclusive if the mother reported only giving the child breast milk throughout the first 6 months of life and no other source of milk (i.e. formula).

### Statistical Analysis

Evaluating fractional polynomial smoothed plots of peak weight and height velocity the first 36 months of life with risk of the respiratory disorders of interest, there was no strong indication of any nonlinear associations. We examined the associations of one standard deviation (SD) increase in peak weight and height velocity with child respiratory disorders by log-binomial regression, reporting relative risks (RR) and 95% confidence intervals (CI). To account for siblings, we used cluster variance estimations. Multivariable regression analysis was used to calculate adjusted RR (adj.RR), adjusting for all potential confounders identified. To examine whether the associations of peak weight and height velocities with child respiratory disorders was mediated through the child’s BMI at the time of disease classification, we adjusted for this covariate in a second multivariable model. We further evaluated potential effect modification of preterm birth and intrauterine growth on the associations by including product terms in the multivariable models. Preterm birth was classified as being born before 37 gestational weeks. Intrauterine growth was classified as the birth weight being small (< 10^th^ percentile), normal (10^th^–90^th^ percentile) and large (> 90^th^ percentile) for gestational age. The amount of missing information for individual covariates was generally low (< 2%). However, we conducted multiple imputation using chained equations, imputing a total of 20 datasets. The results from the multiple imputation analysis are provided in the tables in the paper while the results from the complete case analysis are provided in S1 Table. To assess selection bias due to non-response to follow-up questionnaires we used inverse probability weighting. The weights were the probability of having the necessary follow-up information among eligible children. As a secondary analysis, we performed a paired analysis of siblings who were discordant for the respiratory disorders using conditional logistic regression, reporting odds ratios (OR) and 95% CI. The statistical significance level was 5% for all tests and comparisons. All analyses were conducted in STATA version 13 (Statacorp, Texas).

## RESULTS

**Figure 1:**
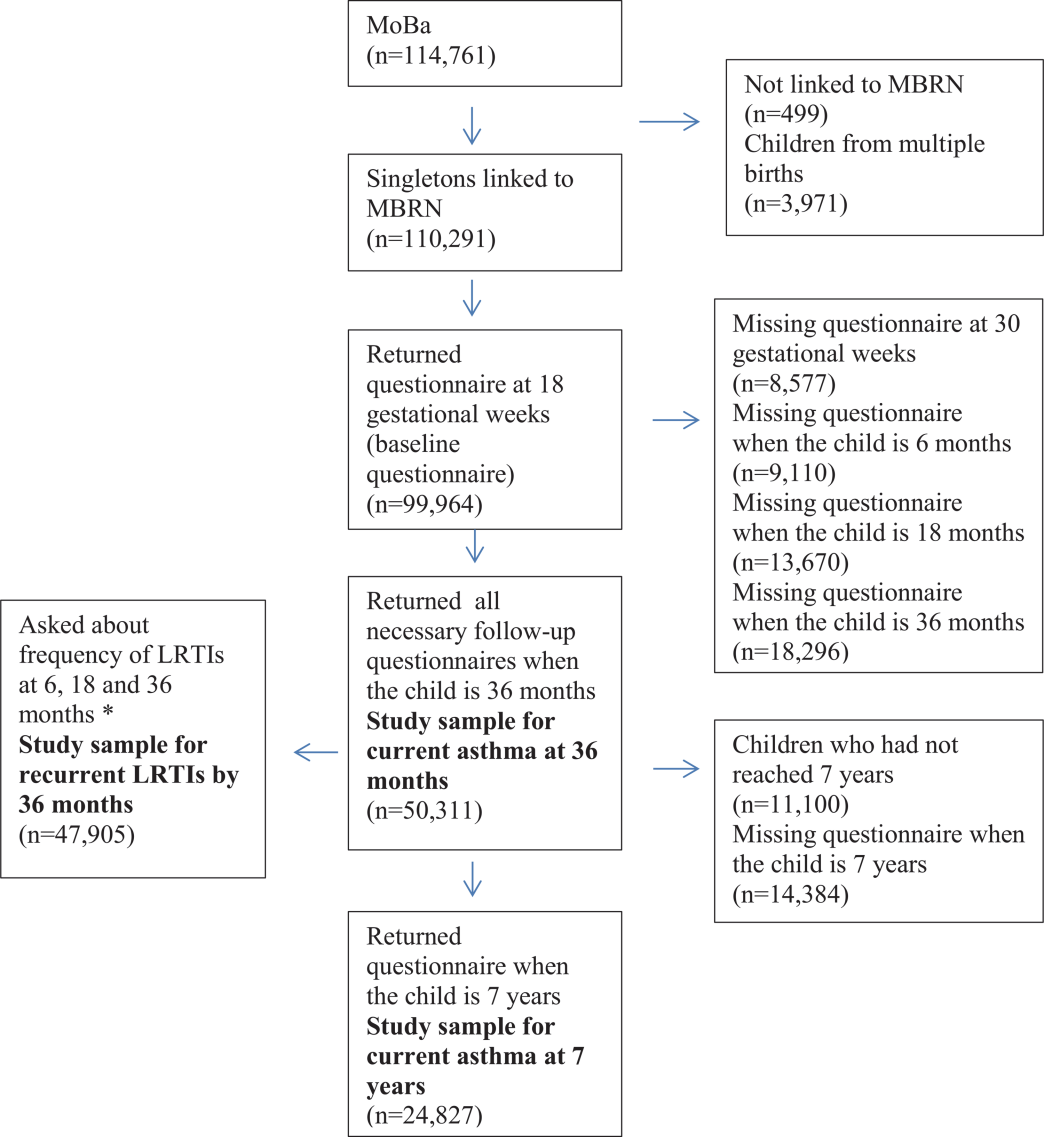
Illustration of sample size. LRTIs- lower respiratory tract infections. ^a^Some versions of questionnaires did not ask about frequency/number of LRTIs.

Of the 110,291 eligible children, 50,311 children were included in the analysis of current asthma at 36 months while 47,905 children were included in the analysis of recurrent LRTIs by 36 months (Fig. 1). Among the 50,311 children in the analysis of current asthma at 36 months, a total of 39,211 children had reached age 7, out of which 24,827 had follow-up information from the 7 year questionnaire and were included in the analysis of current asthma at 7 years (Fig. 1). Characteristics were similar in the sample for current asthma at 36 months and 7 years (Table 1). The mean peak weight velocity was 15.1 kg per year (SD 6.4 kg). The mean age at peak weight velocity was 14 days, where 30.4% had peak weight velocity after birth. The mean peak height velocity was 62.0 cm per year (SD 15.2). The mean age at peak height velocity was 8 days, where 15.4% had peak height velocity after birth. Among children who experienced peak weight velocity after birth, there was a higher proportion of girls, children born preterm and a higher proportion of children born small for gestational age. Similar characteristics were seen for children who experienced their peak height velocity after birth. The correlation between peak weight and height velocity with gestational age, birth weight and height, in addition to later anthropometric measurements are provided in S2 Table.

A total of 5.7% of children had current asthma at 36 months, 4.3% of children experienced recurrent LRTIs by 36 months and 5.1% had current asthma at 7 years. Peak weight velocity was positively associated with current asthma at 36 months, adj. RR 1.22 (95% CI: 1.18, 1.26) per SD increase, recurrent LRTIs by 36 months, adj. 1.14 (95% CI: 1.10, 1.19) per SD increase, and current asthma at 7 years, adj. RR 1.13 (95% CI: 1.07, 1.19) per SD increase (Table 2). These positive associations between peak weight velocity and respiratory disorders remained after additional adjustment for the child’s BMI development. Peak height velocity was not associated with any of the respiratory disorders.

**Table 2:** Associations of one standard deviation increase in peak weight and height velocity the first 36 months of life with development of respiratory disorders.

**Outcome**	**Sample size**	**Exposure**	**Crude RR (95% CI)**	**Adj. RR (95% CI)^a^**	**Adj. RR (95% CI)^b^**
Current asthma at 36 months	50,311	Peak weight velocity	1.24 (1.20, 1.28)	1.22 (1.18, 1.26)	1.24 (1.19, 1.28)
		Peak height velocity	0.97 (0.93, 1.01)	0.97 (0.93, 1.01)	0.97 (0.93, 1.01)
Recurrent LRTIs by 36 months	47,905	Peak weight velocity	1.17 (1.13, 1.22)	1.14 (1.10, 1.19)	1.14 (1.09, 1.19)
		Peak height velocity	0.98 (0.93, 1.02)	0.96 (0.91, 1.01)	0.96 (0.91, 1.01)
Current asthma at 7 years	24,827	Peak weight velocity	1.14 (1.08, 1.20)	1.13 (1.07, 1.19)	1.13 (1.07, 1.20)
		Peak height velocity	0.98 (0.93, 1.04)	1.01 (0.96, 1.08)	1.01 (0.95, 1.08)

LRTIs- lower respiratory tract infections

^a^Adjusted for maternal age, maternal parity, maternal education, maternal salary, maternal height, maternal pre-pregnancy BMI, paternal height, paternal BMI, maternal folate intake during pregnancy, maternal smoking during pregnancy, maternal asthma, paternal asthma, child gender, gestational age and breast feeding the first 6 months of life.

^b^Additional adjustment for the child’s BMI at 36 months (current asthma at 36 months and recurrent LRTIs by 36 months) or 7 years (current asthma at 7 years).

Multiple imputation of missing covariate information conducted using chained equations. A total of 20 imputed datasets generated for pooled analysis.

The positive association of peak weight velocity with child respiratory disorders tended to be stronger among the 95.5% of children born at term, but the test for multiplicative interaction by preterm delivery was not statistically significant (>0.1). In the stratified analysis by intrauterine growth, there was a tendency for an inverse association between peak height velocity and all the respiratory disorders of interest among the 10.2% of children born small for gestational age (adj. RR ~0.8). However, the tests for multiplicative interaction indicated that the association of peak height velocity with the respiratory disorders was not significantly different between children born small, normal and large for gestational age (all p-values >0.1). The analysis using inverse probability weighting to evaluate selection bias yielded similar results as the un-weighted analysis (S3 Table).

There were 488 sibling pairs discordant for asthma at 36 months. In the sibling pair analysis, peak weight velocity showed a positive association with asthma at 36 months, adj. OR 1.27 (95% CI: 1.04, 1.55) per SD increase (Table 3). There were 370 sibling pairs discordant for recurrent LRTIs. Peak weight velocity showed no significant association with recurrent LRTIs in the sibling pair analysis, adj. OR 0.93 (95% CI: 0.75, 1.17), while peak height velocity showed an inverse association, adj. RR 0.81 (0.66, 0.98) per SD increase (Table 3). There were 127 sibling pairs discordant for current asthma at 7 years. Neither peak weight velocity nor peak height velocity showed a significant association with current asthma at 7 years.

**Table 3:** Sibling pair analysis of the associations of one standard deviation increase in peak weight and height velocity the first 36 months of life with development of respiratory disorders.

**Outcome**	**Number of discordant sibling pairs in analysis**	**Exposure**	**Crude OR (95% CI)**	**Adj OR (95% CI)^a^**	**Adj OR (95% CI)^b^**
Current asthma at 36 months	488	Peak weight velocity	1.36 (1.15, 1.60)	1.27 (1.04, 1.55)	1.26 (1.03, 1.55)
		Peak height velocity	1.14 (0.99, 1.32)	0.96 (0.80, 1.15)	0.96 (0.80, 1.15)
Recurrent LRTIs by 36 months	370	Peak weight velocity	1.06 (0.88, 1.27)	0.93 (0.75, 1.17)	0.94 (0.75, 1.19)
		Peak height velocity	0.92 (0.78, 1.08)	0.81 (0.66, 0.98)	0.81 (0.66, 0.98)
Current asthma at 7 years	127	Peak weight velocity	1.24 (0.92, 1.68)	1.03 (0.68, 1.55)	1.01 (0.66, 1.54)
		Peak height velocity	1.26 (0.97, 1.62)	1.19 (0.82, 1.72)	1.21 (0.83, 1.76)

LRTIs- lower respiratory tract infections

^a^Adjusted for maternal age, maternal parity, maternal education, maternal salary, maternal height, maternal pre-pregnancy BMI, paternal height, paternal BMI, maternal folate intake during pregnancy, maternal smoking during pregnancy, maternal asthma, paternal asthma, child gender, gestational age and breast feeding the first 6 months of life.

^b^Additional adjustment for the child’s BMI at 36 months (current asthma at 36 months and recurrent LRTIs by 36 months) or 7 years (current asthma at 7 years).

Multiple imputation of missing covariate information conducted using chained equations. A total of 20 imputed datasets generated for pooled analysis.

## DISCUSSION

In this large population based study, peak weight velocity was positively associated with current asthma at 36 months, recurrent LRTIs by 36 months and current asthma at 7 years. These positive associations were independent of the child’s BMI development. Peak height velocity showed no clear association with any of the respiratory disorders evaluated. The sibling pair analysis supported the positive association of peak weight velocity with current asthma at 36 months.

Most previous studies of infant growth and asthma evaluated change in standard deviation scores or absolute change in weight between two measurement points [11,15–18]. One study evaluated conditional weight gain during infancy [14]. Our findings support previous studies indicating that weight increase the first 3 to 6 months is positively associated with asthma while length increase during this age period shows no association [11,14]. The recent meta-analysis further showed a positive association of absolute weight gain between birth and 12 months of age with asthma development [19].

Only two previous studies used a longitudinal data analysis to model the child’s growth. One study used a multilevel model with restricted cubic splines, reporting a positive association of weight increase the first 12 months with asthma [12]. A German study previously examined peak weight and height velocity using the Reed1 model and development of asthma [13]. In accordance with our study, they reported a positive association between peak weight velocity and asthma development during the first 10 years of age, adjusted hazard ratio 1.22 (95% CI: 1.02–1.47) per interquartile increase, and no association between peak height velocity and asthma development, adjusted hazard ratio 1.08 (95% CI: 0.88, 1.31) per interquartile increase. In our study, we found a similar association between peak weight velocity and recurrent LRTIs by 36 months.

Early infant growth might plausibly have a direct effect on lung development. Human lung development has five overlapping stages that begins at approximately 3 weeks’ post-conception and extends into the second year of life [22]. Alveologenesis starts around 29 gestational weeks and continues after birth. This is therefore the stage of lung development during which the peak weight and height velocity was evaluated. Lung development is influenced by genes, transcription factors, growth factors and cytokines [22].

Intrauterine growth restriction often results in impaired lung development, poorer infant lung function and postnatal catch-up growth [40,41]. Smaller weight gain between the second and third trimester has been positively associated with peak weight velocity as calculated using the Reed model [28]. In addition, gradual length gain between second and third trimester was positively associated with peak height velocity [28]. We only saw an association of peak weight velocity with respiratory disorders while we saw no association with peak height velocity. The positive association between peak weight velocity and childhood respiratory disorders might partly reflect intrauterine growth. However, we found no difference in the association between peak weight velocity and respiratory disorders among children born small, normal and large for gestational age.

Another possible explanation for the association between peak weight velocity and childhood respiratory disorders includes genetic and/or epigenetic mechanisms. One of the most replicated loci in genome wide association studies of childhood asthma development is the 17q12-21 loci [42]. The effect of the 17q12-21 loci is enhanced by fetal and infant smoke exposure [43]. In the MoBa cohort, both prenatal smoke exposure and birth weight is associated with the child’s cord blood DNA methylation pattern [24,44]. Prenatal smoke exposure is a common risk factor for low birth weight, which influences postnatal growth, in addition to asthma development. Therefore, common genetic and/or epigenetic mechanisms might underlie the association between peak weight velocity and childhood respiratory disorders.

There might also be common environmental factors influencing prenatal/postnatal growth and childhood respiratory disorders. Infant feeding is such an example [26,27]. Environmental toxins might also constitute common predictors for postnatal growth and childhood respiratory disorders. Maternal PCB level during pregnancy has been associated with both birth weight and asthma development [45,46]. A variety of environmental factors might therefore influence early postnatal growth and development of childhood respiratory disorders underlying the observed positive association.

MoBa provided a unique opportunity to examine the research question of interest due to its size, availability of 10 anthropometric measurement points, ability to adjust for a large number of potential confounding factors, and the siblings participating in the cohort. Results from several previous studies, the current study, and the meta-analysis largely point in the same direction. We supplement the existing literature by showing that the previously observed associations are most likely not due to factors that previous studies could not adjust for, including paternal asthma and anthropometric values. Furthermore, we found that peak weight velocity by 36 months was also positively associated with recurrent LRTIs. Interestingly, peak weight velocity showed a strong correlation with absolute weight increase from birth to 12 month (correlation coefficient 0.76). Our findings and those from the recent meta-analysis might therefore reflect the same phenomenon. This is the first study to use a sibling pair analysis when examining the association of growth and respiratory disorders. The sibling pair analysis yielded similar associations of peak weight and height velocity with asthma at 36 months. This was not the case for the other two respiratory outcomes. However, we remain cautious in our interpretation of the sibling pair analysis for asthma at 7 years due to the few number of discordant sibling pairs.

The current study has limitations. Maternal report of anthropometric measurements may have caused misclassification. By asking the mother to refer to the child’s health report card, we attempted to minimize this. Furthermore, the Reed1 growth model smoothes the growth curves over the entire growth period and might therefore miss extreme growth changes over very short time periods. Asthma among preschool age children is often characterized by transient wheezing due to respiratory tract infections. In MoBa, 40% of children had experienced wheezing symptoms the previous year when they were 18 months of age. This is substantially lower than the prevalence of asthma at 36 months and might indicate that Norwegian mothers are not reporting all early wheezing symptoms as asthma. Notably, adjusting the association of peak weight and height velocity with asthma development for LRTIs, ear infections and throat infections did not change the results. The asthma classification used in the current study might also be considered fairly stringent compared to the classification used by the global imitative for asthma of wheezing symptoms the past 12 months. A similar asthma case definition as the one used in the current study was validated among 5 year old children in a Finish study population against registered use of asthma medications indicating a high validity [47]. Maternal report of the child’s use of asthma medications the last year in the 7 year questionnaire in MoBa also showed a high validity against registered use of asthma medications in the Norwegian Prescription Database [48]. Excluding the use of asthma medications from the definitions yielded a prevalence of current asthma at 36 months of 6.4% and a prevalence of current asthma at 7 years of 5.2%. Notably, these less stringent asthma definitions yielded the same associations with peak weight and height velocity. Differential misclassification of respiratory disorders is unlikely due to the prospective data collection. Any misclassification would therefore bias the results towards the null. A selection bias might have occurred due to the initial participation rate into MoBa. Comparing MoBa participants to all pregnant women who gave birth during the MoBa inclusion period as registered in the MBRN indicated that MoBa participants were older, less likely to be single, less likely to have more than two previous deliveries and less likely to smoke during pregnancy [31]. However, these differences were not found to influence exposure outcome associations. Overall, we do not think that the initial participation rate is a strong source of selection bias in the current study. However, we cannot exclude the possibility that the differences identified between MoBa participants and all pregnant women registered in the MBRN might reduce the generalizability of the study results with regard to certain underrepresented high-risk groups. The current study further required that the mothers had responded to several follow-up questionnaires. However, the results of the analysis using inverse probability weighting to make the study population more comparable to all eligible MoBa participants yielded similar results. We therefore do not think that the observed associations are strongly influenced by selection bias.

In conclusion, higher peak weight velocity, achieved during the immediate postnatal period, increased the risk of respiratory disorders. This might be explained by an influence on neonatal lung development, shared genetic/epigenetic mechanisms and/or environmental factors.

## Supporting Information

S1 Table Association per standard deviation increase in peak weight and height velocity the first 36 months of life with childhood respiratory disorders: results from complete case analysis. (DOCX)

S2 Table Spearman rank correlation coefficients between peak weight and height velocity the first 36 months of life with gestational age and all anthropometric measurements. (DOCX)

S3 Table Association per standard deviation increase in peak weight and height velocity the first 36 months of life with childhood respiratory disorders: results using inverse probability weighting. (DOCX)

S1 Text Description of growth curve modelling. (DOCX)

S1 Fig Directed acyclic graph. Child characteristics: child gender and breastfeeding the first 6 months. Maternal characteristics: maternal age, maternal education, maternal salary, maternal parity, maternal smoking during pregnancy and maternal folate intake during pregnancy. Genes: Genetic predisposition for growth and asthma. Measured by maternal height, maternal body mass index, paternal height and paternal body mass index, in addition to maternal and paternal history of asthma.
(TIF)
