# Supplementary material for: Peak Weight and Height Velocity to Age 36 Months and Asthma Development: The Norwegian Mother and Child Cohort Study
Source: PLoS One. 2015 Jan 30;10(1):e0116362. doi: 10.1371/journal.pone.0116362 (PMC4312021; doi:10.1371/journal.pone.0116362)
Supplement: S1 Table — (DOCX) [file pone.0116362.s001.docx]

Table S1 Associations of one standard deviation increase in peak weight and height velocity the first 36 months of life with childhood respiratory disorders: results from complete case analysis

| Outcome | Exposure | Crude RR (95% CI) | Adj. RR (95% CI) ^a^ | Adj. RR (95% CI) ^b^ |
| --- | --- | --- | --- | --- |
| Current asthma at 36 months  (N=50,311) | Peak weight velocity | 1.24 (1.20, 1.28) | 1.23 (1.18, 1.28) | 1.25 (1.19, 1.30) |
|  | Peak height velocity | 0.97 (0.94, 1.01) | 0.96 (0.91, 1.01) | 0.95 (0.90, 1.00) |
| Recurrent LRTIs by 36 months  (N= 47,905) | Peak weight velocity | 1.17 (1.13, 1.22) | 1.14 (1.08, 1.20) | 1.11 (1.05, 1.18) |
|  | Peak height velocity | 0.98 (0.94, 1.03) | 0.95 (0.90, 1.01) | 0.95 (0.89, 1.02) |
| Current asthma at 7 years  (N= 24,827) | Peak weight velocity | 1.14 (1.08, 1.20) | 1.13 (1.06, 1.21) | 1.14 (1.07, 1.22) |
|  | Peak height velocity | 0.98 (0.93, 1.04) | 1.01 (0.94, 1.09) | 1.03 (0.95, 1.11) |

^a^Adjusted for maternal age, maternal parity, maternal education, maternal salary, maternal height, maternal pre-pregnancy BMI, paternal height, paternal BMI, maternal folate intake during pregnancy, maternal smoking during pregnancy, maternal asthma, paternal asthma, child gender, gestational age and breast feeding the first 6 months of life.

^b^Additional adjustment for the child’s BMI at 36 months (current asthma at 36 months and recurrent LRTIs by 36 months) or 7 years (current asthma at 7 years).
