# Supplementary material for: Peak Weight and Height Velocity to Age 36 Months and Asthma Development: The Norwegian Mother and Child Cohort Study
Source: PLoS One. 2015 Jan 30;10(1):e0116362. doi: 10.1371/journal.pone.0116362 (PMC4312021; doi:10.1371/journal.pone.0116362)
Supplement: S2 Table — (DOCX) [file pone.0116362.s002.docx]

Table S2 Spearman rank correlation coefficients between peak weight and height velocity the first 36 months of life with gestational age and all anthropometric measurements

| Correlation coefficients with peak weight velocity | Gestational age | 0.07 |
| --- | --- | --- |
|  | Birth weight | 0.20 |
|  | Weight at 6 weeks | 0.51 |
|  | Weight at 3 months | 0.76 |
|  | Weight at 6 months | 0.87 |
|  | Weight at 8 months | 0.86 |
|  | Weight at 12 months | 0.82 |
|  | Weight at 15-18 months | 0.74 |
|  | Weight at 18 months | 0.69 |
|  | Weight at 24 months | 0.58 |
|  | Weight at 36 months | 0.44 |
| Correlation coefficients with peak height velocity | Gestational age | 0.24 |
|  | Birth height | 0.24 |
|  | Height at 6 weeks | 0.60 |
|  | Height at 3 months | 0.59 |
|  | Height at 6 months | 0.45 |
|  | Height at 8 months | 0.33 |
|  | Height at 12 months | 0.22 |
|  | Height at 15-18 months | 0.15 |
|  | Height at 18 months | 0.08 |
|  | Height at 24 months | 0.14 |
|  | Height at 36 months | 0.29 |
