# Supplementary material for: Peak Weight and Height Velocity to Age 36 Months and Asthma Development: The Norwegian Mother and Child Cohort Study
Source: PLoS One. 2015 Jan 30;10(1):e0116362. doi: 10.1371/journal.pone.0116362 (PMC4312021; doi:10.1371/journal.pone.0116362)
Supplement: S1 Text — (DOCX) [file pone.0116362.s004.docx]

Text S1 Description of growth curve modelling

Growth is rapid during infancy and gradually decreases and becomes relatively constant during late preschool years. Fractional polynomial functions are a good method to model growth when the child’s size increases monotonically and the slope of the growth curve decreases monotonically. Several fractional polynomial models have previously been evaluated to model growth among the children in the Norwegian Mother and Child Cohort Study, including the Karlberg model, the Count model and the Reed first-and second-order models [33-35]. These well-known models are fractional polynomial models including age(t) using different combinations of t, t2, 1/t and ln(t) to model human growth. Based on a comparison of model fit, including the -2 log likelihood and the Bayesian Information Criterion, the first order reed model was found to have the best fit when modelling childhood growth among MoBa children [36]. In our study, parametric growth curves for the child’s growth the first 36 months were fitted using the first order Reed model for each gender (boys and girls) separately using the random effects models [33-35]. The best-fitted curves for each individual were estimated using maximum likelihood estimation. Multilevel mixed effects linear regression analysis was used to fit the model using the MIXED option in STATA 13. The random components in this model allows for individual variation in the child’s starting measurements (intercept) and rate of growth (slope). We included random effects for all four components in the Reed1 model when calculating the height velocity, whereas we included random effects for all components except for C when computing the weight velocity. The unstructured variance-covariance option was used to estimate the variance-covariance components. This option allows variance estimates to be distinct, unlike the independent or identity option that confine that variance-covariance components to particular values or patterns.

Reed1 model

The Reed1 model is a 4-paramter extension of the 3-parameter Count model and its functional form is [33]:

Y= A + Bt + Cln(t) + D/(t)

Since this model is not defined at birth (t=0), the model specification was modified to allow for the inclusion of the anthropometric measurements at birth as proposed by Simondon [35]:

Y=A + Bt + Cln(t+30) + D/(t+30), where

t=postnatal age in days

Y=height or weight reached at time t

And A, B, C and D are the functional parameters.

Of the functional parameters, A is related to the baseline height/weight at birth, B is the linear component for the growth velocity, C is related to the decrease in growth velocity over time, while D reflects the inflection point that allows growth velocity to peak after birth rather than exactly at birth.

Since only having one or two anthropometric measurements available was not sufficient to capture the shape of the growth curve, the analysis was restricted to children with a minimum of three anthropometric measurements available.

The individual velocity curves, calculated by the formula dy/dt=B+C/(t+30) - D/ (t+30)^2,^  were then used to obtain biologically meaningful parameters, including peak, minimum and mean height and weight velocities.

As these three biological parameters are strongly correlated, we had a priori decided to evaluate the peak weight and height velocities in relation the respiratory outcomes of interest.
