# Supplementary figures and images for: Peak Weight and Height Velocity to Age 36 Months and Asthma Development: The Norwegian Mother and Child Cohort Study
Source: PLoS One. 2015 Jan 30;10(1):e0116362. doi: 10.1371/journal.pone.0116362 (PMC4312021; doi:10.1371/journal.pone.0116362)

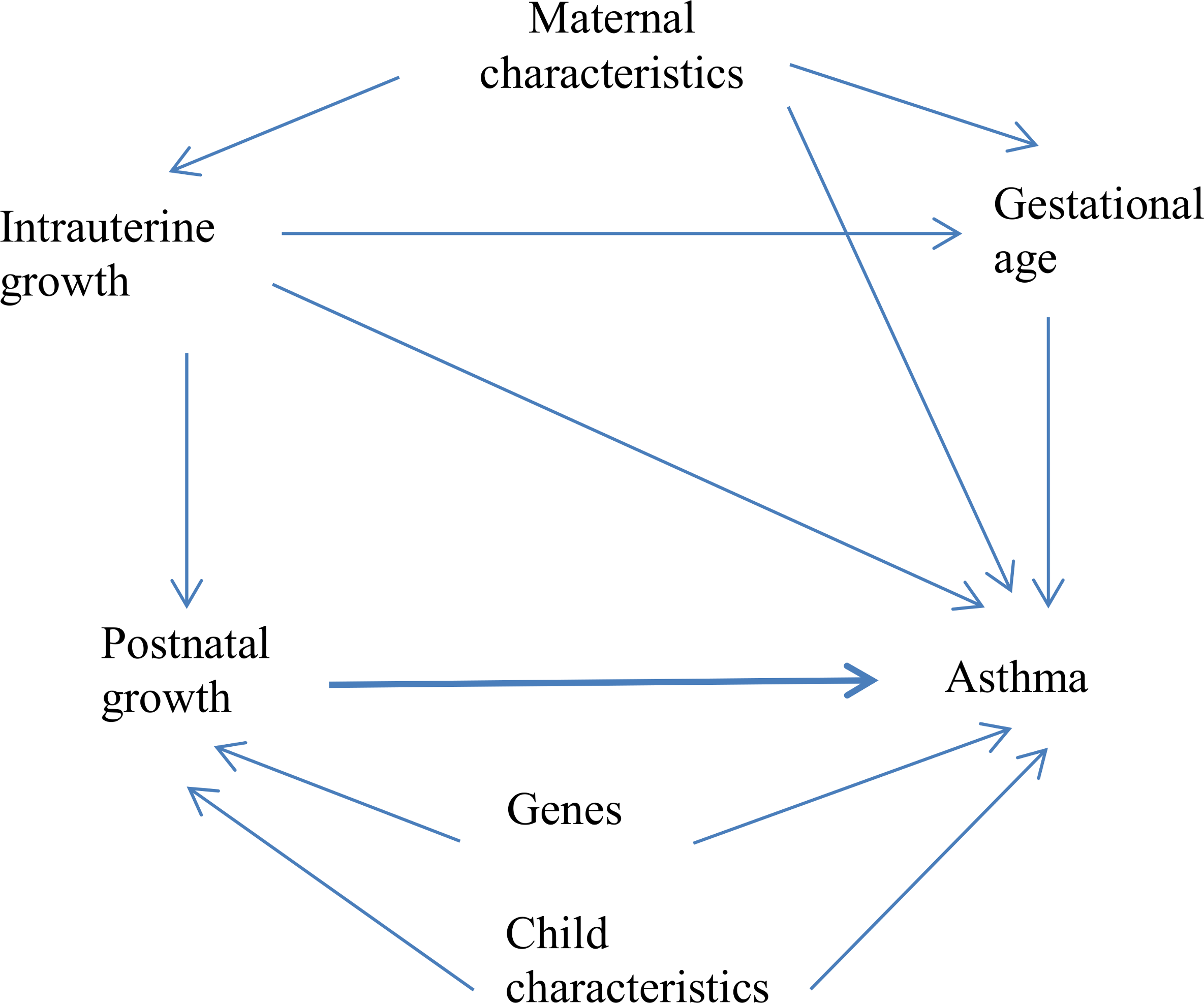

Supplement: S1 Fig — Child characteristics: child gender and breastfeeding the first 6 months. Maternal characteristics: maternal age, maternal education, maternal salary, maternal parity, maternal smoking during pregnancy and maternal folate intake during pregnancy. Genes: Genetic predisposition for growth and asthma. Measured by maternal height, maternal body mass index, paternal height and paternal body mass index, in addition to maternal and paternal history of asthma. (TIF) [file pone.0116362.s005.tif]
